# Tension-band high-strength suture combined with absorbable screws with cortical penetration for treating Mayo type IIA olecranon fractures: finite element analysis, biomechanical testing, and clinical study

**DOI:** 10.3389/fbioe.2026.1840855

**Published:** 2026-05-20

**Authors:** Shunjie Dang, Haoyang Chen, Jie Liang, Jinhui Liu, Wenxun Jiang, Defang Cui, Guiwen Luo, Yunkang Yang, Feifan Xiang

**Affiliations:** 1 Department of Orthopedics, The Affiliated Hospital of Southwest Medical University, Luzhou, China; 2 Sichuan Provincial Laboratory of Orthopaedic Engineering, Luzhou, China; 3 Department of Orthopedics, People’s Hospital of Xuyong County, Luzhou, China

**Keywords:** absorbable screw, biomechanics, clinical study, finite element analysis, high-strength suture, olecranon fracture

## Abstract

**Objective:**

Tension-band wiring with Kirschner wires (TBW) is a common surgical technique for olecranon fractures (OFs), but has limitations like implant removal and soft tissue irritation. Tension-band high-strength suture with absorbable screw (TBSAS) technique can effectively solve these issues. This study aims to demonstrate the biomechanical stability and clinical efficacy of the TBSASC technique for treating OFs (Mayo IIA) (AO 21-B1.1).

**Methods:**

Four finite element models for OFs (Mayo IIA) compared fixation constructs: TBW with cortical penetration (TBWC), TBW with intramedullary fixation (TBWM), TBSAS with cortical penetration (TBSASC), and TBSAS with intramedullary fixation (TBSASM). Displacement and stress distributions were analyzed under various loading conditions. Corresponding 3D-printed physical models tested static tensile strength, fatigue resistance, and torsional stability. Clinically, 13 patients with OFs (Mayo IIA) treated with TBSASC between April 2023 and April 2025 were prospectively followed. Operative data, complications, fracture healing, range of motion, VAS pain score, and MEPS were recorded to assess safety and efficacy.

**Results:**

Finite element analysis (FEA) showed the TBSASC group achieved mean fracture displacements slightly inferior but without statistical significance to the gold-standard TBWC group (max 0.192 mm vs. 0.178 mm, p > 0.05). TBSASC significantly reduced stress on both implants (max 79.99 MPa) and bone (85.80 MPa) compared to TBWC (464.82 MPa and 137.54 MPa, respectively). Biomechanically, TBSASC withstood physiological loads without failure, with its ultimate load, fatigue, and torsional resistance slightly inferior but without statistical significance to TBWC (p > 0.05). Clinically, 13 patients treated with TBSASC were followed for a mean of 14.7 months. Mean operative time was 66.1 min. Fractures healed by 7.1 weeks. Pain (VAS) decreased from 6.0 at 1 month to 0.15 at 6 months, and elbow function (MEPS) improved from 79.2 to 95.8 over 12 months, with 76.9% of patients very satisfied. No complications occurred.

**Conclusion:**

The TBSASC technique demonstrates acceptable preliminary safety and feasibility for OFs (Mayo IIA), providing sufficient stability for fracture fixation and early functional exercise, effectively reducing complications associated with metal implants and trauma from secondary removal surgery, suggesting its potential as a promising alternative that warrants further investigation in larger, prospective comparative studies.

## 1 Introduction

Olecranon fractures (OFs) are common adult fractures, accounting for 10% of all upper extremity fractures and representing the most common type of elbow fracture (Giardina et al., 2024). They are most commonly observed in young patients following high-energy trauma or in elderly individuals after low-energy falls. As intra-articular fractures, OFs typically require surgical treatment. The goals of surgical intervention are to restore elbow joint function and stability, enabling early mobilization while minimizing associated complications (Yin et al., 2023).

Although several classification systems exist for OFs, the most commonly used is the Mayo Classification, which incorporates displacement, comminution, and elbow instability. Mayo Type I fractures are nondisplaced and stable, and may be either noncomminuted (IA) or comminuted (IB). Mayo Type II fractures are displaced but stable, and may be either noncomminuted (IIA) or comminuted (IIB). Mayo Type III fractures are displaced and unstable, and may be either noncomminuted (IIIA) or comminuted (IIIB). Recent studies indicate that up to 85%–90% of OFs are displaced, stable injuries (Mayo Type IIA) (Nowotny et al., 2019). The generally accepted criterion for nonoperative management of fracture displacement is less than 2 mm of articular step-off on a lateral elbow radiograph; otherwise, surgical treatment is indicated (Duckworth et al., 2023).

There are multiple surgical options for OFs (Mayo IIA), including tension-band wiring with Kirschner wires (TBW), proximal ulnar plate (PUP) fixation, and all-suture fixation, among others (Duckworth et al., 2023; Avisar et al., 2024). Among the various available methods, TBW remains one of the most commonly used techniques for treating OFs (Mayo IIA). It is relatively simple and effective, designed to convert the tensile force generated by the triceps muscle into compressive force at the fracture site (Jia et al., 2025). Although most patients achieve satisfactory healing rates and elbow function following TBW fixation (Civan et al., 2021), However, postoperative complications associated with this technique include implant failure, Kirschner wire migration, fracture nonunion, skin and soft tissue irritation, and ulnar nerve injury (Du et al., 2022). Koroglu et al. (Köroğlu et al., 2024) reported a rare case of Kirschner wire distal migration into the forearm 9 months after TBW fixation following olecranon osteotomy, indicating that the wire-cable system fixation at the olecranon is not absolutely stable and carries potential long-term risks. Furthermore, both techniques require a secondary surgery for implant removal, thereby increasing patient discomfort and financial burden (Phadnis et al., 2020).

In recent years, non-metallic implants have garnered significant attention and achieved rapid advancements, leading to new treatment options for fractures and soft tissue injuries in orthopedics. Poly lactic-co-glycolic acid (PLGA) is one of the most widely used biodegradable forged composites, offering good bioactivity, biocompatibility, and high mechanical strength (Kruse et al., 2022). PLGA absorbable cannulated screws can directly integrate with bone, completely replacing natural bone, and ultimately hydrolyze into α-hydroxy acids, with complete absorption occurring in approximately 2 years. Additionally, Ultrabraid™ #2 suture is a non-absorbable, high-strength suture that offers significant biomechanical advantages and is widely used in the treatment of tendon and ligament ruptures, meniscal injuries, and fractures (Liu et al., 2020; Taha et al., 2020). Xiang et al. (2024) have successfully applied the tension-band high-strength suture combined with absorbable screw (TBSAS) technique for the treatment of patellar fractures, noting that the tensile force exerted by the quadriceps muscle on the patella is significantly greater than that exerted by the triceps muscle on the olecranon (Fan et al., 2025). Given that the TBSAS technique is capable of withstanding the high tensile forces of the knee extensor mechanism to meet the fixation requirements of patellar fractures, it theoretically should also possess sufficient mechanical strength to resist the distractive stresses generated by the elbow extensor mechanism at the OFs site. To date, there have been no reports on the use of the TBSAS technique for the treatment of OFs, either domestically or internationally. Furthermore, most studies investigating the use of absorbable screws or all-suture techniques alone for OFs lack robust biomechanical and clinical validation.

This study combined FEA, biomechanical testing, and a clinical study to evaluate the TBSAS with cortical penetration (TBSASC) technique for OFs (Mayo IIA). We hypothesized that TBSASC would provide biomechanical stability comparable to conventional TBW, with reduced implant stress and acceptable early clinical outcomes.

## 2 Materials and methods

### 2.1 FEA: establishment of the OF (Mayo IIA)

**FIGURE 1:**
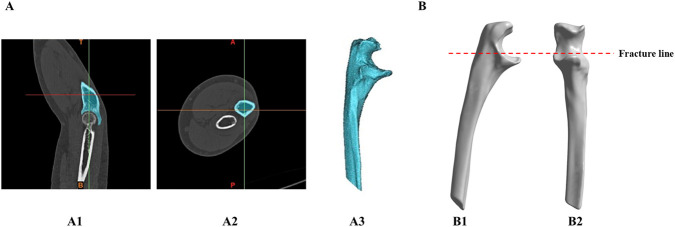
Production of the ulna and internal fixation model: **(A)** 3D ulna model extracted from image data, A1-A2: CT image of the Elbow joint; A3: 3D ulna model. **(B)** Establishing the ulnar olecranon fracture (OF Mayo IIA) model, B1-B2: anteroposterior view and lateral view.

This study was approved by the Medical Ethics Committee of our institution (KY2024466), and all participants provided informed consent. Image data of the olecranon were obtained from a healthy female volunteer (age: 45 years, height: 165 cm, weight: 63 kg) using spiral computed tomography (CT) (128-slice spiral CT scanner, GE Medical Systems; slice thickness: 0.5 mm) and saved in Digital Imaging and Communications in Medicine format. In Mimics Research 21 (Materialize, Belgium), threshold segmentation, region growing, and other commands were used to extract the cortical and cancellous bone structures of the olecranon to construct a three-dimensional model (Figure 1A). Mesh reconstruction, wrapping, and smoothing were performed using Geomagic Wrap 2023 (Geomagic, NC, United States). Finally, the OF (Mayo IIA) model (AO 21-B1.1) was established using SolidWorks 2023 (Dassault, France) (Figure 1B) (Chang et al., 2023).

### 2.2 FEA: establishment of internal fixation models

**FIGURE 2:**
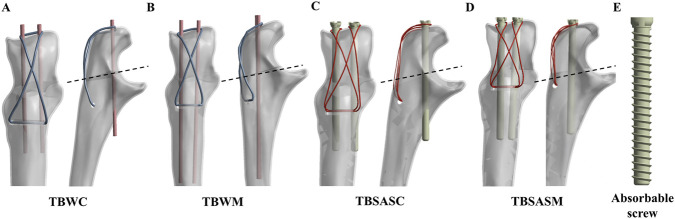
OFs (Mayo IIA) internal fixation assembly model: **(A)** TBW with cortical penetration fixation (TBWC). **(B)** TBW with intramedullary fixation (TBWM). **(C)** TBSAS with cortical penetration fixation (TBSASC). **(D)** TBSAS with intramedullary fixation (TBSASM). (Blue: steel wire; Pink: Kirschner wire; Red: Ultrabraid sutures; Yellow: absorbable screw; Black dotted line: fracture line). **(E)** Absorbable screw.

In this study, four internal fixation methods for OF (Mayo IIA) were selected to compare and investigate the biomechanical effects of the TBSASC group. Fixation was performed using SolidWorks 2023 software based on the OF model and standard surgical protocols (Figures 2A–D). The patients were divided into four groups: TBW with cortical penetration fixation (TBWC), TBW with intramedullary fixation (TBWM), TBSAS with intramedullary fixation (TBSASM), and the experimental group TBSASC. The diameters of the Kirschner wires and steel wire were 2 mm and 1 mm, respectively. Absorbable cannulated screws with a diameter of 4.5 mm and a length of 50 mm were selected (Figure 2E). During numerical analysis, the absorbable screw was simplified to a threadless model to circumvent complex contact convergence difficulties in the calculations, conserve computational resources, and retain the core information of the overall mechanical behavior (Wang et al., 2022). For the high-strength suture, a diameter of 0.58 mm was selected. From the distal to the proximal aspect of the OF, two Kirschner wires or screws were placed parallel using “Boolean operations,” inserted from the posterior articular surface of the proximal ulna through the cortex to the anterior one-third of the proximal ulna (Civan et al., 2021). A transverse bone tunnel was created in the distal ulna, positioned at least 1 cm from the dorsal cortex of the ulna and at least 2–3 cm distal to the fracture site (Duckworth et al., 2023).

### 2.3 FEA: material properties, boundary, and loading conditions

The material properties used in this study were based on previous literature reports parameters for the various materials are listed in Table 1.

**TABLE 1:** Model material parameters.

Material	Young’s modulus (MPa)	Poisson’s ratio
Cortical bone	18000	0.3
Cancellous bone	5000	0.3
Kirschner wire	210000	0.3
Steel wire	10000	0.3
Absorbable screw	5500	0.3
Ultrabraid suture	3000	0.4

**FIGURE 3:**
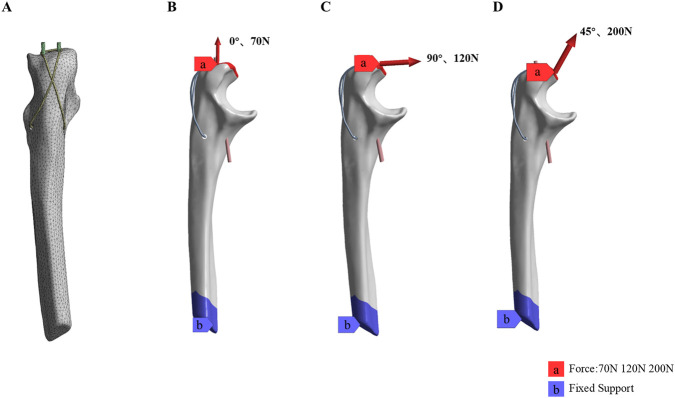
The setting of OF (Mayo IIA) model FEA: **(A)** Model mesh rendering. **(B–D)** Boundary and loading conditions of simulated Elbow joint extension (0°70N load), flexion (90°120N load) and half flexion (45°200N load).

Simulation analysis of the models was performed using ANSYS Workbench 2023 R1 (Swanson Analysis, Houston, PA, United States). The models were meshed using quadratic tetrahedral elements (Figure 3A). A convergence analysis was conducted to ensure the stability and accuracy of the mesh configuration (Huang et al., 2023). The average mesh sizes for the olecranon, Kirschner wires, steel wire, absorbable cannulated screws, and high-strength suture were 0.7 mm, 0.5 mm, 0.5 mm, 0.5 mm, and 0.4 mm, respectively. The models consisted of an average of 1,352,343 nodes and 489,620 elements. Solid 187 tetrahedral element types were selected for the overall solid model. All materials were modeled as homogeneous and linearly isotropic.

To simulate the actual conditions of contact relationships, all contact types were set within the scope of Coulomb’s friction law: bone-bone (friction coefficient μ = 0.45), bone-implant (μ = 0.3), and implant-implant (μ = 0.2) (Chen et al., 2019; Hang et al., 2022). Cortical bone and cancellous bone were modeled as bonded contacts. Based on previous studies, the force and direction of the triceps muscle during elbow extension and flexion were simulated. Within an elbow range of motion of 0°–90°, the triceps muscle was subjected to tensile forces ranging from 0 to 200 N. Specifically, the triceps tensile force was 70 N with the elbow in full extension at 0°, 120 N with the elbow flexed at 90°, and 200 N with the elbow in semi-flexion at 45° (Greenfield et al., 2017). During the analysis, nodes on the distal ulnar surface were constrained with 0 degrees of freedom to simulate the pulling direction of the triceps muscle projecting onto the proximal olecranon and to prevent rigid body motion (Figures 3B–D) (Civan et al., 2021).

### 2.4 Biomechanical testing

**FIGURE 4:**
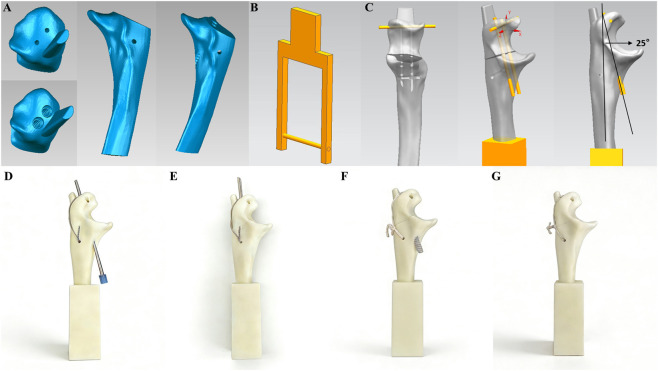
Establishment of biomechanical experimental models and ABS models of four internal fixation for olecranon fracture. **(A)** Schematic diagram of the olecranon model with pre-set 2 mm Kirschner wire track hole diameter and 4.5 mm threaded screw hole. **(B)** Schematic diagram of the high-strength spring steel combined with high-strength tension band bow model. **(C)** Schematic diagram of the pre-set tracks for Kirschner wire insertion angle (25°) and absorbable cannulated screw insertion angle (25°). **(D–G)**: **(D)** 3D-printed ABS model of TBW with cortical penetration fixation (TBWC). **(E)** 3D-printed ABS model of TBW with intramedullary fixation (TBWM). **(F)** 3D-printed ABS model of TBSAS with cortical penetration fixation (TBSASC). **(G)** 3D-printed ABS model of TBSAS with intramedullary fixation.

The models were fabricated using Acrylonitrile Butadiene Styrene (ABS) photosensitive resin with a 3D printing lithography machine (Cong and Zhang, 2025; Fan et al., 2025). High-strength spring steel was inserted into the proximal fracture fragment in combination with a high-strength tension bow to simulate the triceps tendon. Both the Kirschner wire and absorbable cannulated screw channels were pre-defined using SolidWorks 2023 (Figures 4A–C) (Ernstbrunner et al., 2022). The absorbable cannulated screws used in this study were fabricated from Polylactic Acid (PLA) with an outer diameter of 4.5 mm. PLA was selected due to its favorable initial mechanical properties; its flexural strength and elastic modulus are sufficient to meet the stiffness requirements of the implant in vitro biomechanical testing and effectively simulate the mechanical environment during the early stages of fracture fixation, demonstrating good biomechanical suitability (Middleton and Tipton, 2000). According to previous literature, in short-term static and fatigue testing, the difference in initial fixation strength between PLA and the more clinically commonly used PLGA is not clinically significant (Hollinger, 1983; Zhou et al., 2021). All modeling and fixation procedures were performed by the same experienced surgeon, ultimately establishing four groups of fracture-internal fixation physical models (TBWC, TBWM, TBSASC, and TBSASM), with fifteen models in each group (Figures 4D–G).

**FIGURE 5:**
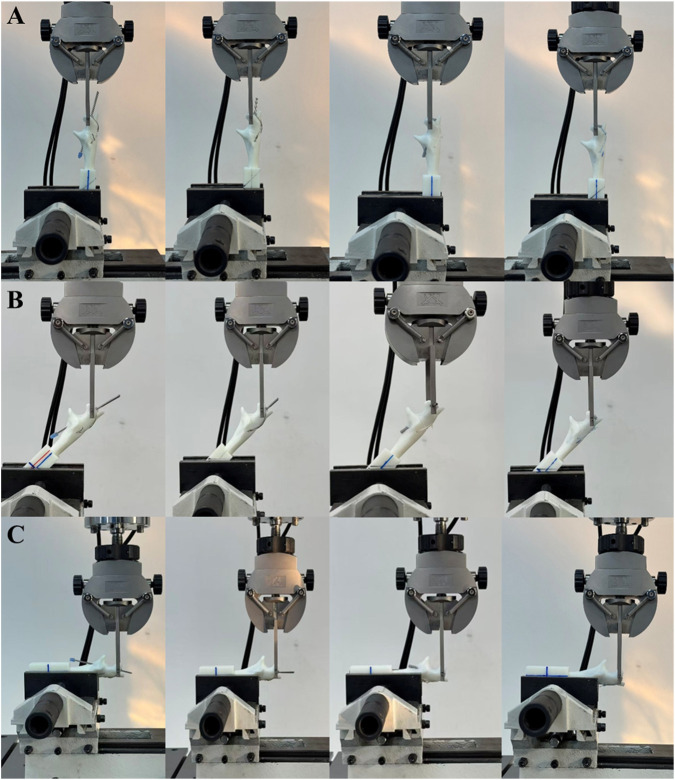
Biomechanical testing of four internal fixation models at three different angles. **(A)** 0° (simulating extension position). **(B)** 45° (simulating semi-flexion position). **(C)** 90° (simulating flexion position).

For static tensile testing, the models (n = 5/group) were secured in an electronic biomechanical testing machine (Instron ElectroPuls E3000 V1.4), pre-tensioned, and constrained at the distal ulna. Tensile load was applied to the proximal olecranon at a set loading rate of 1 mm/min under three physiological angles (0°/70 N, 45°/200 N, 90°/120 N) until the preset tension was reached (Figures 5A–C) (Hutchinson et al., 2003; Calisal and Uğur, 2023). The displacement upon reaching the preset tension and the tension corresponding to displacements of 0.5, 1.0, 1.5, and 2.0 mm were recorded. If the 2 mm failure threshold was not reached upon achieving the preset tension, loading was continued until either failure occurred or displacement reached 2 mm. The critical tension value was recorded, and force-displacement curves were plotted to assess stability (Zhang et al., 2023). For dynamic fatigue testing, models (n = 5/group) prepared using the same method were subjected to 1000 cycles of loading at a frequency of 4 Hz under loads of 40 ± 30 N at 0°, 120 ± 80 N at 45°, and 75 ± 45 N at 90°. Durability was evaluated by analyzing the cycle-displacement curves (Midtgaard et al., 2020; McKnight et al., 2025).

For torsion testing, models (n = 5/group) (Figures 5A–C) were subjected to a torsional load from 0° to 10° at a constant angular velocity of 2.0°/min. Torque-angle curves were recorded, and the critical torque and angle at failure or the maximum torque at 10° were observed to evaluate torsional resistance (Edwards et al., 2012).

### 2.5 Clinical study

Clinical and follow-up data were collected from 13 patients with OF treated using the TBSASC technique at two hospital campuses between April 2023 and April 2025. All participants provided informed consent. Inclusion criteria were: 1) Diagnosis of (Mayo IIA) confirmed by CT or X-ray; 2) Fresh closed fracture (within 3 weeks); 3) Underwent the TBSASC surgical procedure; 4) Age between 18 and 80 years; 5) Provided informed consent and had complete clinical data. Exclusion criteria were as follows: 1) Other types of OFs or open fractures; 2) OFs secondary to infection, tumor, or metabolic disease; 3) Severe structural damage around the olecranon; 4) Poor compliance, failure to undergo follow-up as required, or occurrence of special physiological/pathological changes rendering continued participation unsuitable.

### 2.6 Surgical procedure

**FIGURE 6:**
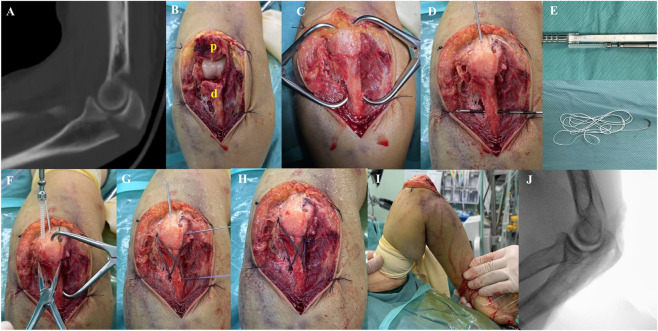
Intraoperative application of TBSASC for the treatment of (Mayo IIA). **(A)** Preoperative CT image; **(B)** Exposure of the fracture site (p: proximal ulna; **(D)** distal ulna); **(C)** Fracture reduction; **(D)** Establishment of transverse bone tunnel; **(E)** Absorbable cannulated screw and Ultrabraid high-strength suture; **(F)** Two 4.5 mm absorbable cannulated screws; **(G)** Figure-of-eight fixation with double-strand high-strength suture; **(H)** Post-fixation status; **(I)** Passive flexion and extension of the elbow joint; **(J)** Postoperative X-ray image.

All surgeries were performed by the same experienced trauma orthopedic surgeon. After induction of anesthesia, the patient was placed in the supine position. A tourniquet was applied to the root of the upper arm on the affected side to control bleeding in the surgical area. The operative field was routinely disinfected, and sterile drapes were applied to prepare for the procedure. A posterior midline elbow incision, approximately 5 cm in length, was made to expose and clear the fracture ends. A transverse bone tunnel was drilled at least 2–3 cm distal to the fracture site and at least 1 cm anterior to the dorsal cortex using a 2.0 mm K-wire. The fracture was temporarily reduced using pointed reduction forceps. After confirming the smoothness of the articular surface, guide pins were inserted, and two 4.5 mm absorbable screws were implanted. Double-stranded Ultrabraid high-strength suture was passed through the distal transverse bone tunnel. When passing the suture through the proximal ulna, it was necessary to grasp the triceps tendon deeply and securely. The suture was then tightened and fixed using tension band and “NICE” knot techniques (Figure 6). Passive flexion and extension of the elbow joint were performed intraoperatively to check the stability of the fracture fixation and elbow motion. Intraoperative data, including operative time, blood loss, and incision length, were recorded. Of note, to ensure rigorous postoperative management, we enhanced dressing changes and focused on wound recovery. Imaging examinations were performed immediately postoperatively. Postoperative plaster fixation was not required; instead, an adjustable elbow brace was worn to assist functional exercise. Flexion and extension training of the elbow joint began on the first postoperative day, and partial weight-bearing activities were gradually introduced. Strenuous exercise was avoided for the first 3 months postoperatively. At 1 month postoperatively, the target range of motion was 90°.

### 2.7 Postoperative data collection

Data on the injury mechanism, time from injury to surgery, operative duration, incision length, and blood loss were recorded. Subsequently, imaging examinations of the affected limb were evaluated immediately postoperatively and at 1 month, 3 months, 6 months, 1 year, and 2 years post-surgery. Data including elbow range of motion, Mayo Elbow Performance Score (MEPS), Visual Analogue Scale (VAS) pain score, postoperative complications, and patient satisfaction were recorded. Fracture healing was assessed based on imaging findings and clinical outcomes, guiding patients in elbow functional exercises.

### 2.8 Outcome measures

For the FEA, the primary outcome measures were olecranon displacement and mean relative displacement of the fracture fragments, with secondary outcome measures including von Mises stress in implants and bone. For the biomechanical testing, the primary outcome measure was ultimate load to failure, and secondary outcome measures were fatigue resistance and torsional stiffness. For the clinical study, the primary outcome measure was fracture healing time, and secondary outcome measures included MEPS, VAS score, range of motion, operative time, incision length, blood loss, complications, and patient satisfaction.

### 2.9 Statistical analysis

SPSS 29 and Prism 10.0 software were used for statistical analysis. Continuous data were tested for normality using the Shapiro-Wilk test. Data conforming to a normal distribution were expressed as mean ± standard deviation, while data not conforming to a normal distribution were expressed as median (interquartile range). For the FEA (mean fracture displacement) and biomechanical testing components, one-way ANOVA was used to compare differences between groups, with a significance level set at P < 0.05. Dunnett’s *post hoc* test was performed following ANOVA. For the clinical component (non-comparative case series, n = 13), only descriptive analysis (mean, standard deviation, frequency, percentage) was performed.

## 3 Results

### 3.1 FEA: displacement of fractures

**FIGURE 7:**
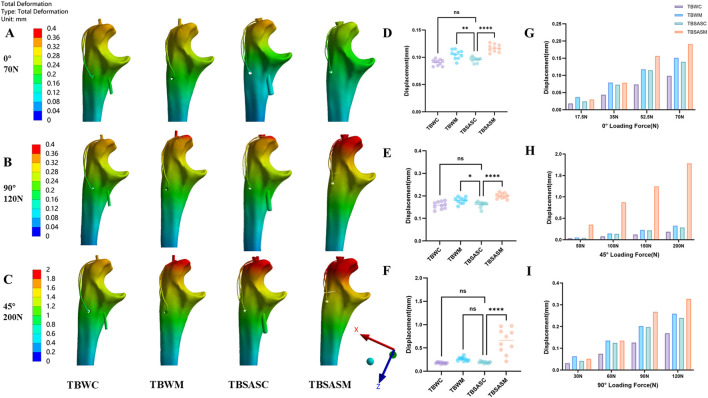
Displacements of the four models under 0°,90°,45° loads: **(A–C)** Displacement nephograms of the four models under 0° load of 70N, 90° load of 120N and 45° load of 200N; **(D–F)** Statistical graphs of average fracture interface relative displacement of the four models under 0° load of 70N, 90° load of 120N and 45° load of 200N; **(G–I)** Maximum bone displacements of the four models under 0° load of 70N, 90° load of 120N and 45° load of 200N.

We calculated the mean fracture gap displacement at 10 points with the same mesh number and the maximum displacement under three loading conditions: 70 N at 0°, 120 N at 90°, and 200 N at 45° (Figure 7). The results are shown in the maximum displacement nephograms for the four model groups under the three loading conditions (Figures 7A–C). Regarding mean fracture gap displacement, except for the TBSASM group, the mean displacement of the fracture fragments was relatively small in the remaining three model groups. Under the three loading conditions mentioned above, the mean fracture gap displacement in the TBWC group (0.091 mm, 0.159 mm, 0.179 mm) was smaller than that in the TBSASC group (0.096 mm, 0.162 mm, 0.192 mm). However, no statistically significant difference was observed between TBWC and TBSASC (Figures 7D–F). Except under the 200 N load at 45°, where no statistically significant difference in mean fracture gap displacement was observed between TBWM and TBSASC (Figure 7F), TBSASC showed statistically significant differences compared to the TBWM and TBSASM groups under the remaining loading conditions. Notably, in the TBSASM group under the 200N load at 45°, the mean fracture gap displacement was relatively large (0.649 mm), reaching a maximum of 0.972 mm. Displacement in all four groups increased with increasing load, with the maximum displacement occurring under the 200 N load at 45° in the following ascending order: TBWC (0.187 mm), TBSASC (0.288 mm), TBWM (0.325 mm), TBSASM (1.783 mm). Compared to the TBSASC technique, the TBWC technique exhibited a 34.87% reduction in maximum displacement under the maximum load of 200 N (0.187 mm vs. 0.288 mm). Compared to the TBWM technique, the TBSASC technique showed a 13.14% reduction in maximum displacement under the maximum load of 200 N (0.288 mm vs. 0.325 mm) (Figures 7G–I).

### 3.2 FEA: stress distribution on internal fixation and olecranon

**FIGURE 8:**
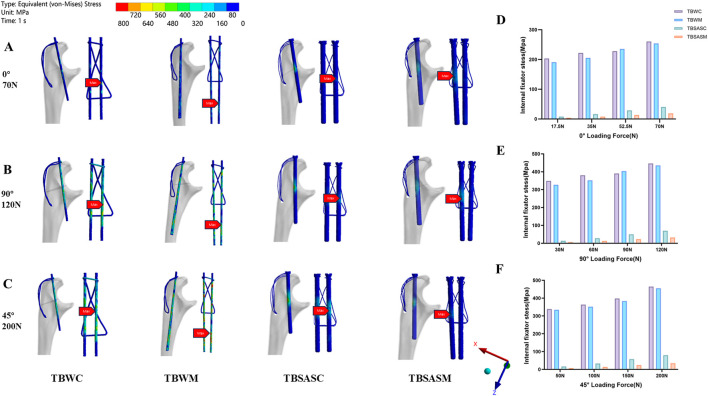
Stress distribution on internal fixation of the four models under 0°,90°,45° loads: **(A–C)** Stress distribution on internal fixation of the four models under 0° load of 70N, 90° load of 120N and 45° load of 200N; **(D–F)** The maximum stresses on internal fixation of four models under 0° load of 70N, 90° load of 120N and 45° load of 200N.

**FIGURE 9:**
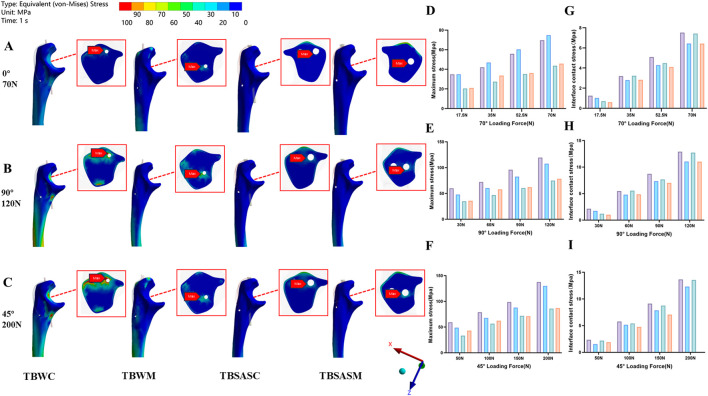
Stress distribution on olecranon of the four models under 0°,90°,45° loads: **(A–C)** Stress distribution on olecranon of the four models under 0° load of 70N, 90° load of 120N and 45° load of 200N; **(D–F)** The maximum stresses on olecranon of four models under 0° load of 70N, 90° load of 120N and 45° load of 200N. **(G–I)** The maximum fracture interface contact stress of four models under 0° load of 70 N, 90° load of 120 N and 45° load of 200 N.

The maximum stress values and stress distribution for each group under different loading conditions are shown in Figure 8 (internal fixation system) and Figure 9 (olecranon and fracture surface). The results indicated that, under all loading conditions, the maximum stresses on both the internal fixation and the olecranon in the TBSASC technique group were lower than those in the other groups.

Under the three loading conditions, except for the TBWM group, stress concentration in the internal fixation of the other three groups occurred mainly at the fracture site (Figure 8). The maximum von Mises stress on the internal fixation for the four model groups decreased in the following order under the three loading conditions: TBWC (260.51 MPa, 446.50 MPa, 464.82 MPa), TBWM (254.21 MPa, 435.71 MPa, 455.50 MPa), TBSASC (40.74 MPa, 69.83 MPa, 79.99 MPa), TBSASM (19.08 MPa, 32.71 MPa, 34.65 MPa). Stress distribution on the olecranon was primarily concentrated at the contact areas with the internal fixation (Figure 9). Under 70 N at 0°, the maximum von Mises stress from highest to lowest was (Figures 9A,D): TBWM (74.878 MPa), TBWC (69.674 MPa), TBSASM (45.78 MPa), and TBSASC (43.69 MPa). Under 120 N at 90°, the order from highest to lowest was (Figures 9B,E): TBWC (119.42 MPa), TBWM (107.69 MPa), TBSASM (74.88 MPa), and TBSASC (78.47 MPa). Under 200 N at 45°, the order from highest to lowest was (Figures 9C,F): TBWC (137.54 MPa), TBWM (129.93 MPa), TBSASC (85.80 MPa), and TBSASM (86.77 MPa). Regarding contact stress at the fracture interface (Figures 9G,H), all three fixation techniques except TBSASM generated favorable interface compression. Progressive separation was observed in the TBSASM group, which was more pronounced under the 200N load at 45°, where the compressive stress was 0.

Compared to the TBWC and TBWM techniques, the TBSASC technique reduced the maximum stress on the olecranon under the 200N load by 37.62% (137.54 MPa vs. 85.80 MPa) and 33.97% (129.93 MPa vs. 85.80 MPa), respectively, while the maximum stress on the internal fixation was reduced by 82.79% (464.82 MPa vs. 79.99 MPa) and 82.44% (455.52 MPa vs. 79.99 MPa), respectively.

### 3.3 Biomechanical testing results: static tensile testing

**FIGURE 10:**
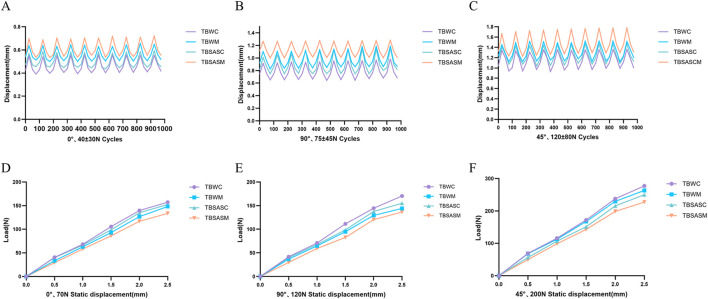
Schematic diagram of cyclic fatigue and static tensile biomechanical test results. **(A–C)** Schematic diagrams of cyclic fatigue test results at 0°, 90°, and 45°, respectively. **(D–F)** Schematic diagrams of static tensile test results at 0°, 90°, and 45°, respectively.

Static tensile testing results (Figure 10; Table 2) demonstrated that TBSASC exhibited good resistance to displacement in all three directions (0°, 90°, and 45°), with overall stability slightly inferior to TBWC but superior to TBWM and TBSASM. The fracture gap displacement recorded for TBSASC upon reaching the preset tension was 1.14 ± 0.01 mm, 1.76 ± 0.03 mm, and 1.91 ± 0.02 mm, respectively. Except for the displacement at 45° being slightly lower than that of TBWM, TBSASC was generally superior to TBSASM (0°: 1.31 ± 0.03 mm; 90°: 1.98 ± 0.05 mm; 45°: 2.02 ± 0.03 mm, p < 0.05) and TBWC (0°: 1.21 ± 0.02 mm; 90°: 1.79 ± 0.02 mm, p < 0.05). Furthermore, at angles of 0°, 90°, and 45°, the ultimate load for TBSASC at 2 mm displacement was 135.10 ± 0.96 N, 137.8 ± 1.02 N, and 215.6 ± 1.38 N, respectively. Again, except for the ultimate load at 2 mm displacement at 45° being slightly lower than that of TBWM, TBSASC was generally superior to TBSASM (0°:116.81 ± 1.19 N; 90°:120.2 ± 0.91 N; 45°: 198.6 ± 1.32 N, p < 0.05) and TBWM (0°:126.82 ± 1.12 N; 90°:129.4 ± 0.93 N, p < 0.05). TBSASC exhibited the optimal load performance at 0° and 90°, significantly better than TBWM and TBSASM, and slightly weaker than TBWC.

**TABLE 2:** Results of biomechanical experiments. All data are shown as mean (SD) unless otherwise stated.

Group	Static (N, mm)			Load at 2 mm gap/N			Fatigue (1,000 cycles, mm)		
	0° 70N	90° 120N	45° 200N	0°	90°	45°	40 ± 30N	75 ± 45N	120 ± 80N
TBWC	1.07 (0.02)	1.62 (0.03)	1.74 (0.03)	139.71 (1.03)	144.5 (0.97)	237.5 (1.25)	0.46 (0.06)	0.77 (0.11)	1.16 (0.14)
TBWM	1.21 (0.02)	1.79 (0.02)	1.83 (0.03)	126.82 (1.12)	129.4 (0.93)	229.7 (1.30)	0.56 (0.05)	0.96 (0.11)	1.30 (0.15)
TBSASC	1.14 (0.01)	1.76 (0.03)	1.91 (0.02)	135.10 (0.96)	137.8 (1.02)	215.6 (1.38)	0.49 (0.05)	0.86 (0.13)	1.26 (0.15)
TBSASM	1.31 (0.03)	1.98 (0.05)	2.02 (0.03)	116.81 (1.19)	120.2 (0.91)	198.6 (1.32)	0.60 (0.07)	1.13 (0.11)	1.47 (0.19)

### 3.4 Biomechanical testing results: dynamic fatigue testing

Dynamic fatigue testing results (Figure 10; Table 2) showed that under a load level of 40 ± 30 N, after 1000 cycles, the TBWC group exhibited the most stable performance with a cumulative displacement of 0.46 ± 0.06 mm. Displacement in the TBSASC group under this load was 0.49 ± 0.05 mm, slightly higher than TBWC, but with no statistically significant difference between the two (p > 0.05). Displacement in the TBWM and TBSASM groups was 0.56 ± 0.05 mm and 0.60 ± 0.07 mm, respectively, with TBSASC showing a statistically significant difference compared to TBWM (p < 0.05) and an even more significant difference compared to TBSASM (p < 0.01). When the load increased to 75 ± 45 N, displacement increased correspondingly in all groups, but a similar trend was maintained. Displacement in the TBWC group was 0.77 ± 0.11 mm, and in the TBSASC group was 0.86 ± 0.13 mm, with no statistically significant difference between them. However, the TBWM and TBSASM groups exhibited displacements of 0.96 ± 0.11 mm and 1.13 ± 0.11 mm, respectively, significantly higher than the first two groups. In the test at the highest load level (120 ± 80 N), the differences between groups became more pronounced. The TBWC group still maintained the lowest displacement (1.16 ± 0.14 mm), while the TBSASC group had a displacement of 1.26 ± 0.15 mm. Although the difference between these two reached statistical significance (p < 0.05), the numerical difference was only 8.6%. In contrast, displacement in the TBWM and TBSASM groups reached 1.30 ± 0.15 mm and 1.47 ± 0.19 mm, respectively, with the cumulative displacement in the TBSASM group being 16.7% higher than that in the TBSASC group. Observing the cumulative displacement curves (Figures 2, 3) across the three load levels, the fatigue resistance of the TBSASC group exhibited two important characteristics: first, its rate of displacement increase with cycling was most similar to that of the TBWC group; second, at all load levels, displacement in the TBSASC group was significantly lower than in the TBSASM group (p < 0.01); and in most cases superior to or equivalent to the TBWM group, with no statistically significant difference compared to TBWC (p > 0.05).

Furthermore, following static tensile testing under three physiological loads and 1000 cycles of dynamic fatigue testing, no implant failure or breakage occurred in any group except for TBSASM, which exhibited gap displacement reaching 2 mm upon reaching the preset tension of 200 N at 45°. In all other groups, displacement did not exceed the OF failure threshold (2 mm) at any of the three angles.

### 3.5 Biomechanical testing results: torsion testing

**FIGURE 11:**
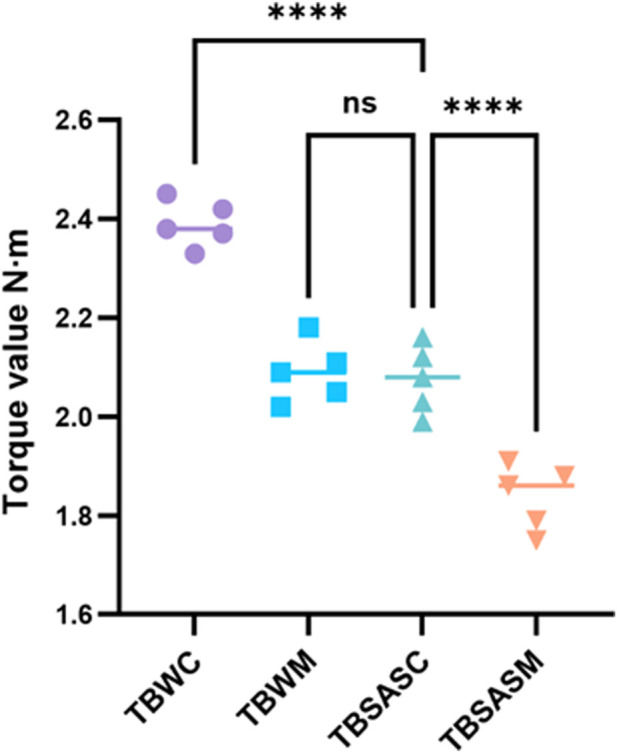
Statistical diagram of torque results for four groups of internal fixation specimens at a torsion angle of 10°.

Torsion testing results (Figure 11; Table 3) showed that the torque value for TBSASC upon reaching 10° of torsion was 2.22 ± 0.07 N m. Although this was slightly lower than the gold standard TBWC (2.29 ± 0.05 N m), the difference was not statistically significant (p > 0.05). Furthermore, TBSASC was significantly superior to TBWM (2.11 ± 0.05 N m) and TBSASM (1.84 ± 0.06 N m), with both differences reaching statistical significance (p < 0.05).

**TABLE 3:** Torque values (N m) of four groups of specimens at a torsion angle of 10°.

Group	Torque value 1 (N)	Torque value 2 (N)	Torque value 3 (N)	Torque value 4 (N)	Torque value 5 (N)	Mean ± SD (N· m)
TBWC	2.23	2.31	2.35	2.32	2.22	2.29 ± 0.05
TBWM	2.12	2.18	2.05	2.09	2.11	2.11 ± 0.05
TBSASC	2.13	2.21	2.18	2.26	2.32	2.22 ± 0.07
TBSASM	1.86	1.79	1.91	1.88	1.75	1.84 ± 0.06

### 3.6 Clinical study results

**FIGURE 12:**
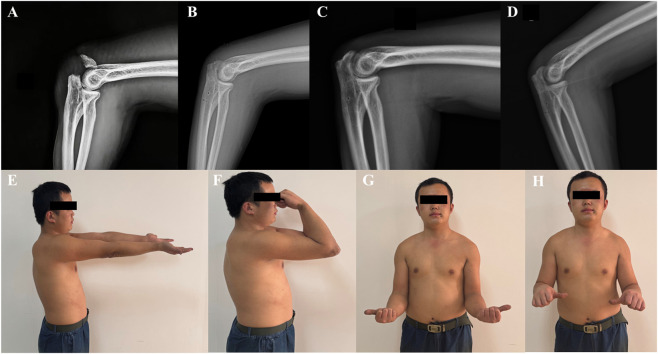
Typical case: A 24-year-old male with right OF (Mayo IIA) caused by a fall. **(A)** Preoperative imaging; **(B)** One month postoperatively; **(C)** Three months postoperatively; **(D)** Nine months postoperatively; **(E–H)** Functional photographs at 1 year postoperatively.

A total of 13 patients with OFs (Mayo IIA) were enrolled in this study according to the inclusion and exclusion criteria, including 5 males and 8 females, with a mean age of 49.38 ± 19.10 years. Causes of injury included falls in 9 cases, high falls in 2 cases, motor vehicle accident in 1 case, and physical injury in 1 case. The mean operative time for all patients was 68.08 ± 7.10 min, intraoperative blood loss was 86.15 ± 24.34 mL, and the mean incision length was 7.23 ± 1.59 cm. Patients were discharged within 3–5 days postoperatively and received rigorous follow-up and guided functional exercises (Table 4). The 13 patients in the TBSASC group were followed for a mean of 14.69 ± 2.02 months. Fracture healing time was 7.08 ± 1.93 weeks. Regarding postoperative pain, the VAS score decreased from 6.00 (moderate) at 1 month postoperatively to 1.62 (mild) at 3 months, and approached zero (0.15) at 6 months, suggesting a mild postoperative pain response and a shorter recovery period in patients treated with TBSASC. The mean MEPS scores at 3, 6, and 12 months postoperatively were 79.23, 82.69, and 95.77, respectively. At the final follow-up, 76.9% of patients were “very satisfied” with their elbow function, and the remaining 23.1% were “satisfied” (Table 5). Regarding complications, all 13 patients in the TBSASC group had well-healed incisions with no signs of redness, swelling, or skin irritation. A typical case is shown in Figure 12.

**TABLE 4:** Baseline characteristics of the enrolled patients.

Patient	Age (years)	Gender	Fracture side	Injury mechanism	Prep-time (days)	Surgery duration (min)	Blood loss (mL)	Incision length (cm)	Follow-up (months)
1	38	Male	Right	Tumble	5	65	60	5	18
2	53	Female	Left	Fight	4	70	90	8	17
3	24	Male	Right	Tumble	7	62	60	6	17
4	63	Female	Right	Tumble	3	65	80	10	16
5	20	Female	Right	Tumble	3	70	90	5	16
6	68	Female	Right	High fall	5	55	60	9	15
7	54	Female	Right	Tumble	5	70	70	7	15
8	35	Male	Left	Tumble	6	75	100	7	14
9	46	Male	Right	Tumble	4	70	100	6	13
10	80	Female	Right	High fall	6	65	80	8	13
11	71	Female	Left	Tumble	7	78	100	8	13
12	29	Female	Right	Car accident	3	80	150	6	12
13	61	Male	Right	Tumble	5	60	80	9	12

Prep-time represents Pre-operative preparation time.

**TABLE 5:** Clinical data of the enrolled patients.

Patient	VAS score	VAS score	VAS score	Mayo score	Mayo score	Mayo score	Final ROM	Time to clinical union (weeks)	Final-satisfaction
	1	3	6	3	6	12			
1	6	2	0	85	85	100	145	6	3
2	7	3	0	80	85	95	135	4	2
3	5	2	0	85	85	100	135	8	3
4	6	2	0	70	75	85	140	8	2
5	7	1	1	80	85	95	140	6	3
6	6	2	0	85	85	100	130	8	3
7	5	1	0	85	85	100	135	10	3
8	6	1	0	80	80	90	130	8	3
9	6	2	0	70	80	95	145	6	2
10	7	1	1	80	85	100	140	10	3
11	5	2	0	70	75	90	135	8	3
12	6	1	0	80	85	95	130	4	3
13	6	1	0	80	85	100	140	6	3

VAS, Score 1, Score 3, and Score 6 represent the VAS, scores at 1 month, 3 months, and 6 months postoperatively, respectively; Mayo score 1, Score 6, and Score 12 represent Mayo scores 1 month, 6 months, and 12 months postoperatively, respectively; MEPS, score: Excellent: 90 points or more, good: 75–89 points, medium: 60–74 points, difference: less than 60 points; ROM: elbow range of motion; Clinical healing criteria for fracture: absence of local tenderness and longitudinal percussion pain, no abnormal mobility, X-ray showing blurred fracture line with continuous callus bridging the fracture gap, and ability to lift 1 kg weight with the upper limb for 1 min or walk continuously without crutches for 3 min (no less than 30 steps) after removal of external fixation. Satisfaction: 0 points: dissatisfied, 1 point: unsure, 2 points: satisfied, 3 points: very satisfied.

## 4 Discussion

The olecranon constitutes an important part of the ulnohumeral joint of the elbow and serves as the insertion point for the triceps muscle, making it crucial for maintaining elbow extension strength and stability. OFs fall into the category of osteoporotic fractures, and an increase in their incidence can certainly be anticipated in the coming decades. As these fractures are inherently intra-articular, the ultimate goal of treatment is to restore joint function. Anatomical reduction, preservation, and early mobilization are recommended as the current standard for treating almost all proximal ulnar fractures, yielding the best outcomes (Šafran et al., 2025). In recent years, significant progress has been made in the treatment of OFs, particularly in the areas of individualized surgical planning guided by classification systems, the development and application of novel internal fixation materials, the promotion of minimally invasive surgical techniques, and the establishment of systematic rehabilitation concepts.

The TBW technique is considered the gold standard surgical procedure for treating OF (Mayo IIA). Previous literature reports that while TBW achieves satisfactory clinical outcomes in terms of postoperative elbow range of motion and Mayo Elbow Performance Score (MEPS) for OFs, related complications arising from Kirschner wires and steel wire cannot be ignored due to the superficial location of the olecranon. These complications include implant irritation, postoperative pain, delayed wound healing, and the need for secondary implant removal, with a reoperation rate reported as high as 71.7% (Wang and Li, 2025; Zhang et al., 2025; Baidya et al., 2026 the efficacy of four different surgical techniques (plate-screw fixation, intramedullary screw fixation, K-wire tension band wiring, and all-suture fixation) for the treatment of OFs. Their results showed that although the K-wire tension band group exhibited poorer outcomes regarding implant loosening, reoperation rate, and radiographic healing delay, overall, all four techniques provided satisfactory postoperative and patient-reported outcomes. This suggests that surgeons can choose their preferred approach based on their own technical expertise and individual patient factors. This conclusion supports the safety foundation of the TBSASC technique we have adopted.

Some scholars have adopted suture techniques as an alternative. Suture techniques reduce the need for implant removal and lower the reoperation rate, resulting in cost savings (Jia et al., 2022). These techniques primarily include suture bridge fixation, bone tunnel fixation using simple or flat braided sutures, suture anchor fixation, and transosseous high-strength suture fixation for the treatment of OFs (Dogramatzis et al., 2023). Awad et al. (2025) treated 25 patients with simple or minimally comminuted OFs using suture tension band fixation. The results showed that no patient required secondary surgery due to implant prominence or failure, and a mean elbow arc of motion of 134° with good functional scores was achieved at the final follow-up. Similarly, Rodà et al. (2025) in a study of 34 patients, also confirmed that the suture tension band technique is a reliable alternative for treating simple OFs, with low complication and reoperation rates. Bateman et al. (2015) successfully treated OFs using double-row suture anchors, with all 8 patients achieving uneventful healing in an acceptable position without displacement. Phadnis and Watts (2017) demonstrated that following isolated high-strength suture fixation for OFs, there were no reoperations or wound complications, and all treated patients achieved good or excellent functional outcomes with minimal loss of motion. However, most studies investigating the use of absorbable screws or suture techniques alone for the treatment of OFs lack robust finite element, biomechanical, and clinical trial validation.

Currently, there are no reports on the use of the TBSAS technique for the treatment of OFs, either domestically or internationally. Furthermore, Xiang et al. (2024) in their use of the TBSAS technique for treating patellar pole fractures, demonstrated that the fixation construct exhibited strong rigid fixation strength, providing enhanced stability and reducing the risk of fracture displacement. This finding provides an important reference for extending the application of the TBSAS technique to fractures in other similar anatomical sites. From a biomechanical perspective, the patella and olecranon are both integral components of the extensor mechanism. Given that the TBSAS technique is sufficient to meet the fixation requirements of patellar fractures, the mechanical environment that fracture fixation must resist is similar. Previous literature indicates that under normal conditions, the tensile force exerted by the quadriceps muscle on the superior pole of the patella is significantly higher than that exerted by the triceps muscle on the proximal olecranon (Fan et al., 2025). If the TBSAS technique is capable of withstanding the high tensile forces of the knee extensor mechanism to meet the fixation requirements of patellar fractures, then theoretically, it should also possess sufficient mechanical strength to resist the distractive stresses generated by the elbow extensor mechanism at the OF site. Therefore, we attempted to apply the tension-band high-strength suture combined with absorbable cannulated screw (TBSAS technique) to the treatment of (Mayo IIA). This technique, by simulating and modifying the mechanical principles of traditional tension band wiring (TBW) and combining absorbable screws with high-strength sutures using tension band and “NICE” knot connection techniques, could theoretically further enhance the compression fixation of OFs. The aim is to address the challenge of minimizing complications such as soft tissue irritation while ensuring adequate internal fixation strength, and simultaneously meeting the requirements for early functional exercise of the elbow joint.

Our study primarily evaluated the safety and efficacy of the TBWC and TBSASC techniques for the treatment of OFs (Mayo IIA). The results demonstrated that with the TBSASC technique, under the three applied loading conditions, the displacement and stress on the olecranon and implants were relatively small, and satisfactory compression was achieved at the fracture site. Furthermore, during clinical follow-up, satisfactory clinical outcomes were obtained in terms of elbow range of motion and elbow function scores, with high patient satisfaction, good fracture healing, and no requirement for secondary implant removal.

In the FEA regarding fracture site stability, except for the TBSASM group, the other three model groups exhibited relatively small mean fracture gap displacements under the three loading conditions, indicating a generally good ability to maintain fragment alignment and resist shear and separation forces. Notably, the mean fracture gap displacements of the TBWC and TBSASC groups were the most similar, with no statistically significant difference, suggesting that these two techniques may possess comparable efficacy in controlling micromotion at the fracture site. It is worth noting that the TBSASM group showed a significant increase in mean displacement under the high load of 200 N at 45°, with a maximum value reaching 0.972 mm, indicating potential instability of this fixation method when resisting larger combined loads and a risk of gap formation at the fracture site. This finding corroborates the results of the fracture interface contact stress analysis: in the TBSASM group, the interface compressive stress decreased to 0 under high load, indicating that the fixation failed to maintain effective compression at the fracture site, potentially leading to a suboptimal healing environment. In contrast, the other three groups all exhibited sustained interface compressive stress, which is conducive to close fracture fragment apposition and early healing.

The stress distribution within the internal fixation system is directly related to its risk of fatigue fracture. The internal fixation in the TBWC and TBWM groups experienced significantly higher von Mises stresses compared to the TBSASC and TBSASM groups, with stress values exceeding 450 MPa under maximum load. Stress concentration occurred primarily at the fracture site, approaching or potentially exceeding the fatigue limit of some metallic materials, suggesting a higher likelihood of implant failure under cyclic loading. In contrast, the maximum stress values in the TBSASC and TBSASM groups were substantially lower, indicating that these constructs may more effectively distribute load or transfer it through the bone fragments themselves, thereby reducing the mechanical burden on the implants and theoretically enhancing the long-term durability of the construct. Similar to TBW, TBSASC minimizes soft tissue damage and promotes fracture healing by avoiding extensive periosteal stripping and disruption of the soft tissues surrounding the fracture (Bethell et al., 2025). The TBSAS tension band construct, similar in configuration to TBW, possesses the ability to convert posterior tensile forces into compressive forces at the joint surface, which can accelerate fracture healing and permit early postoperative rehabilitation (Appleton et al., 2025). Furthermore, due to the specific material properties of the absorbable cannulated screws and high-strength suture, the Young’s modulus of PLGA is closer to that of bone, and the suture material exhibits better elasticity compared to steel wire. Consequently, the internal fixation and ulnar stress results were lower in the TBSAS groups.

The stress distribution within the olecranon bone itself reflects the pattern of load transfer from the internal fixation to the bone. The TBWC and TBWM groups exhibited higher peak stresses on the olecranon, concentrated in local areas in contact with the implants. Such high stress concentrations could potentially increase the risk of postoperative local bone resorption or cut-out. In contrast, the bone tissue stress distribution in the TBSASC and TBSASM groups was more moderate, with significantly reduced peak stresses, demonstrating better load-sharing and load-dissipating characteristics. This may be more favorable for protecting bone tissue and reducing local complications.

Regarding the biomechanical testing, the results of the static tensile and dynamic fatigue loading experiments were highly consistent with the finite element simulation findings. This consistency further validates the accuracy and reliability of the established finite element models, indicating that the finite element models in this study can effectively simulate actual mechanical behavior.

The static tensile test results clearly established the biomechanical profile of each group. First, it must be objectively acknowledged that the ultimate load of the experimental TBSASC group was significantly lower than that of the gold standard TBWC in all three testing directions. This finding directly confirms that, under the test conditions established in this study, TBWC still possesses an irreplaceable advantage in peak strength for resisting maximum tensile loads in a single direction. However, the more important value of this experiment lies in clarifying the “relative superiority” of TBSASC. The data show that TBSASC not only consistently and significantly outperformed the homologous TBSASM construct in all directions, validating the effectiveness of its specific design modifications, but more critically, it surpassed the widely used TBWM in both the 0° and 90° directions. Furthermore, numerous studies indicate that the strength of an implant only needs to meet the biological threshold required for protective healing in the early postoperative period; excessively high stiffness may instead lead to stress shielding, which is detrimental to long-term bone remodeling (Ma et al., 2023). Although the strength and stability provided by TBSASC are lower than those of TBWC, they are still far above the threshold required for physiological demands.

Dynamic fatigue testing simulates the behavior of the joint under long-term cyclic loading and is a key indicator for evaluating the durability of the internal fixation system. TBSASC exhibited minimal cumulative displacement at all three load levels, indicating that the construct maintains good stability under repetitive loading. This suggests that TBSASC possesses superior fatigue resistance when coping with high-intensity exercise or unexpected loads. Fatigue failure is a common postoperative complication of internal fixation, particularly in athletes or active individuals. By enhancing the suture-bone interface integration and optimizing the load transfer pathway, TBSASC effectively delays the accumulation of fatigue damage, potentially extending the service life of the fixation system and reducing the risk of secondary surgery. The results of the torsion testing indicate that TBSASC demonstrates reliable performance in resisting rotational stress, making it suitable for joint functional recovery scenarios requiring resistance to torsional loads. Clinically, joint torsion often accompanies daily activities or sports movements, such as turning or changing direction. Through structural optimization, TBSASC enhances torsional resistance without excessively increasing stiffness, which is conducive to achieving the rehabilitation goal of “stable yet flexible”.

TBSASC achieved satisfactory results in the clinical trial. For intra-articular fractures, although open reduction and internal fixation with plates offers distinct advantages in restoring articular surface congruity and stability, its performance in terms of postoperative elbow range of motion, joint stiffness rate, and fracture healing rate still requires further improvement. While elbow arthroplasty can restore joint function in the short term, it is associated with a high rate of long-term complications and is not suitable for all patients. Therefore, in clinical practice, the optimal treatment plan should be selected by considering factors such as the patient’s age, fracture type, and soft tissue condition (Jia et al., 2025). TBW remains the most commonly used surgical technique for treating OFs, and its application has gradually evolved to include personalized 3D-printed navigation. Given that OFs are inherently intra-articular, meticulous open reduction of the fracture fragments is essential for achieving satisfactory clinical outcomes (Zhang et al., 2025). Compared to TBW, the TBSASC procedure is highly similar in its steps, except for replacing the temporary Kirschner wires with absorbable cannulated screws, making it simple and flexible to perform. The mean operative time, intraoperative blood loss, and incision length of TBSASC fell within the range of previously reported values for TBW (García-Elvira et al., 2020). Clinical follow-up showed that, owing to rigorous wound management and exercise guidance, none of the 13 patients experienced postoperative infection, implant failure, revision surgery, or other serious complications. In the early postoperative period, patients in the TBSASC group reported milder pain and a shorter recovery period, with lower VAS scores, creating favorable conditions for early functional exercise. Notably, all 13 patients achieved elbow flexion greater than 100° at 1 month postoperatively, indicating that TBSASC provides sufficient fixation strength and stability to meet the requirements for OF fixation and early functional exercise. Although the initial surgical cost of this technique is relatively high, it effectively reduces complications associated with metal implants and avoids the trauma and pain of secondary implant removal. Therefore, in this study, more patients tended to prefer treatment with TBSASC.

Overall, the TBWC demonstrated the best performance in maintaining fracture site stability, followed by the TBSASC group; however, the former achieved this at the cost of subjecting the internal fixation to extremely high stress. TBWM generally occupied an intermediate position across various indicators. However, due to the traction force of the triceps muscle, the intramedullary fixation method of TBWM may not provide sufficient internal fixation strength in the early stages, particularly during elbow flexion (Hu et al., 2025).

This study still has several limitations. First, FEA comparing the mechanical trends of various internal fixation constructs involved simplifications of the model structure and loading conditions. Second, the material models used assumed that both cortical and cancellous bone tissues were linearly elastic, isotropic, and homogeneous. While such simplifications are commonly used in FEA for outcome comparisons to evaluate the mechanical interactions and relative performance of fixation constructs, they do not capture the inherent heterogeneity and anisotropy of bone. Furthermore, FEA modeling requires addressing numerous complex details and constraints; our model did not incorporate ligaments or cartilage and thus cannot fully simulate the actual kinematic process of the elbow joint. Additionally, the analysis lacked assessment of different fracture types under various angles. Furthermore, the absorbable screws used in this study were made of PLA, primarily chosen for cost control and preparation efficiency in the experimental setting. In reality, there are differences in degradation rate and biocompatibility between PLA and PLGA; PLA degrades more slowly. Finally, clinical study was a case series design without concurrent control group, with small sample size and short follow-up, risking selection and assessment bias; limited generalizability based on single healthy volunteer CT model and small clinical cohort; TBSASC currently only validated for OFs (Mayo IIA).

In the future, we aim to completely reconstruct a kinematic structural model of the elbow joint, incorporating the joint’s motion cycle into our studies. We will strive to apply the TBSASC technique to a wider range of fracture types. Subsequent animal studies will require validation using PLGA materials with degradation characteristics more suitable for clinical application. With the improvement of clinical patient follow-up, we plan to conduct in-depth biomechanical investigations into screw absorption and fracture healing.

## 5 Conclusion

The TBSASC technique demonstrates biomechanical promise and exhibits preliminary biomechanical feasibility and acceptable early clinical efficacy in small early clinical series. Future controlled comparative studies with larger sample sizes and longer follow-up periods are needed to confirm that it provides sufficient stability for fracture fixation and early functional exercise, with the potential to reduce metal implant-related complications and avoid secondary removal surgery.

## Data Availability

The original contributions presented in the study are included in the article/supplementary material, further inquiries can be directed to the corresponding authors.
